# The relation between psychological profiles and quality of life in patients with lung cancer

**DOI:** 10.1007/s00520-019-04923-w

**Published:** 2019-07-01

**Authors:** Eveline van Montfort, Jolanda de Vries, Rita Arts, Joachim G. Aerts, Jeroen S. Kloover, Marjan J. Traa

**Affiliations:** 1 CoRPS - Center of Research on Psychology in Somatic Diseases, Department of Medical and Clinical Psychology, Tilburg University, Tilburg, The Netherlands; 2 ETZ Hospital (Elisabeth-TweeSteden Ziekenhuis), Tilburg, The Netherlands; 3 Erasmus MC-Cancer Institute, Rotterdam, The Netherlands

**Keywords:** Lung cancer, Psychological profiles, Anxiety symptoms, Depressive symptoms, Quality of life, Coping styles, Perceived social support

## Abstract

**Objective:**

Previous studies in patients with lung cancer examined the association between psychological factors with quality of life (QoL), as well as the association between psychological factors with sociodemographic and medical characteristics. However, knowledge about the impact of combinations of psychological characteristics on QoL is still lacking. Therefore, the current study aimed to identify psychological profiles, covering multiple psychological factors. Additionally, the association between these profiles with QoL and with sociodemographic and medical characteristics was explored.

**Methods:**

Patients with lung cancer (*n* = 130, mean age = 68.3 ± 8.6 years; 49% men) completed questionnaires focusing on sociodemographic information, anxiety and depressive symptoms (HADS), coping (COPE-easy), perceived social support (PSSS), and QoL (WHOQOL-BREF). Medical information was extracted from patients’ medical records. A step-3 latent profile analysis was performed to identify the psychological profiles. Multinomial logit models were used to explore the medical and sociodemographic correlates of the profiles and the relation with QoL.

**Results:**

Four psychological profiles were identified as follows: (1) anxious, extensive coping repertoire (33%); (2) depressive, avoidant coping (23%); (3) low emotional symptoms, active/social coping (16%); and (4) low emotional symptoms, limited coping repertoire (29%). QoL in profile 1 (QoL = 6.59) was significantly different from QoL in profile 3 (QoL = 8.11, *p* = .001) and profile 4 (QoL = 7.40, *p* = .01). QoL in profile 2 (QoL = 6.43) was significantly different from QoL in profile 3 (QoL = 8.11, *p* = .003) and profile 4 (QoL = 7.40, *p* = .02). Regarding QoL, no other significant differences were found. Sociodemographic and medical characteristics were not distinctive for the profiles (all *p* values > .05).

**Conclusion:**

Determining psychological profiles of patients with lung cancer in an early stage provides information that may be helpful in aligning care with patients’ unique needs, as it will help in more adequately selecting those patients who are in need of psychological screening and/or psychological treatment as compared with determining scores on single psychological factors.

## Introduction

Lung cancer is the leading cause of cancer-related mortality worldwide [1]. Lung cancer is frequently an incurable disease with an intensive course of treatment and is associated with greater levels of psychological distress than any other cancer type [2]. The prevalence of depressive symptoms ranges from 25 to 44% and for anxiety from 16 to 43% [3–7]. Sometimes, depressive or anxiety symptoms become a clinical problem that leads to unacceptable suffering, which in turn adversely affects patients’ quality of life (QOL) [6, 8, 9].

Previous studies found that younger age and female sex were positively correlated with higher levels of depressive and anxiety symptoms [9–11]. It was also found that depressive symptoms were more prevalent in patients with small cell lung cancer than in patients with non-small cell lung cancer [4]. However, in another study, this association between cell type and depression was not confirmed [6].

Interestingly, in some cases, specific psychological factors, such as the extent in which patients perceive a stigma, were stronger related to depressive symptoms and QoL as compared with sociodemographic and medical characteristics [12, 13].

Furthermore, relatively stable psychological factors may play an important role in this context [10]. More specifically, patients’ coping styles, which are defined as relatively permanent, individual-specific involuntary behaviors that are used to deal with stressful situations [14], may affect psychological symptoms, and consequently QOL [15]. The efforts patients make to manage their disease and the consequences of the disease may either induce or protect against emotional symptoms. For example, blaming oneself for getting lung cancer may cause emotional symptoms, while seeking social support may protect against these symptoms [10].

The extent of perceived social support may be another factor of interest [16]. Being highly confident about the availability of adequate social support when needed may promote emotional well-being, because social contacts may provide positive experiences. Moreover, the feeling of being close with relatives may reduce the intensity of unpleasant psychological symptoms such as fear or helplessness. In contrast, being less confident about the availability of adequate social support may have an adverse impact on QoL [16].

In sum, previous studies examined the association between psychological characteristics with QoL in patients with lung cancer, as well as the association between psychological factors witch sociodemographic and medical characteristics. However, knowledge about the impact of combinations of psychological characteristics on QoL, which may differ from the impact of single psychological factors, is still lacking. This knowledge gap may partly be explained because previous studies mostly used a *variable-centered* approach, which examined psychological characteristics in isolation from each other. To gain more insight in the association between combinations of psychological factors and QoL, a *person-centered* approach is required. This approach, where the unit of analysis is the patient, studies individual profiles, which cover unique information that is not incorporated by the use of single psychological factors [17, 18]. Identifying psychological profiles, covering multiple psychological characteristics instead of single characteristics may be helpful in identifying patients with a high risk to experience a low QOL, but also in the development of effective and personalized psychological interventions that are tailored to patient’s unique needs [15].

Therefore, the current study applied a *person-centered* approach with the aim to identify latent psychological profiles in patients with lung cancer. These profiles were based on a broad set of psychological characteristics, including transient emotional symptoms (i.e., anxiety and depressive symptoms), relatively stable psychological factors (i.e., coping styles), and the extent of perceived social support. It was also evaluated how the profiles were linked to sociodemographic and medical characteristics. Finally, the link between the profiles and QoL was examined. Three hypotheses were formulated as follows:

- Hypothesis 1: In patients with lung cancer, distinct latent psychological profiles can be identified, based on a broad set of psychological characteristics, including transient emotional symptoms (i.e., anxiety and depressive symptoms), relatively stable psychological factors (i.e., coping styles), and the extent of perceived social support.
- Hypothesis 2: The identified psychological patient profiles are associated with specific sociodemographic and medical characteristics.
- Hypothesis 3: QoL is significantly different between the identified psychological patient profiles.

## Methods

### Participants and design

Patients eligible for inclusion (i) were diagnosed with lung cancer between November 2016 and April 2017 in the ETZ Hospital (Elisabeth-TweeSteden Ziekenhuis), Tilburg, The Netherlands (ii) 18 years or older, (iii) alive, (iv) had sufficient understanding of the Dutch language, and (v) did not have cognitive or psychiatric problems. A researcher explained the study purpose and highlighted that participation was voluntary. Next, patients received an informative letter, the questionnaire, and the consent form. Patients did not receive financial compensation. The study was in accordance with the 1964 Helsinki Declaration and received approval from the regional medical ethical committee (METC/jv/2013.194 protocol no.1373).

### Sociodemographic and medical information

Information on age, sex, marital status, work status, and education level was obtained through the questionnaire. Patient’s medical information was retrieved from the Eindhoven Cancer Registry (ECR). The ECR routinely collects data from patients’ medical records on tumor characteristics including date of diagnosis, tumor grade according to the tumor-node-metastasis medical classification, clinical stage, and treatment. Missing information was retrieved from patient’s medical records.

### Psychological characteristics

Anxiety and depressive symptoms were assessed using the Hospital Anxiety and Depression Scale (HADS) [19]. The HADS comprises of two subscales: one for anxiety and one for depressive symptoms (both 7 items). All items were scored on a Likert scale from 0 (never) to 3 (almost every day). Total scores ranged from 0 to 21, with higher scores indicating higher levels of symptoms. Previously, a threshold of ≥ 8 was identified for optimal balance between sensitivity and specificity [19]. Psychometric properties of this instrument are good [19]. In the current study, internal consistency was high (Cronbach alpha .80 for anxiety and .83 for depressive symptoms).

The COPE-easy was used to measure how patients deal with stressful situations [20]. This instrument is frequently used in cancer research [21, 22]. As it is allowed to select items of specific coping styles, items that were considered unsuitable for the current patient group (e.g., humor, waiting until the right moment) were deleted from the list [20]. This resulted in a 20-item questionnaire, comprising of three coping styles: active coping (8 items, e.g., to make a plan), seeking social support (6 items, e.g., to ask someone for advice), and avoidance coping (6 items, e.g., getting upset). All items were rated on a Likert scale from 1 (not) to 4 (very). Total scores for active coping ranged from 8 to 32, for seeking social support and avoidance from 6 to 24, with higher scores indicating a higher tendency to use this coping style in stressful circumstances. The COPE-easy has adequate validity and reliability [21, 22]. Internal consistency was high in the current study (Cronbach alpha active coping .91, seeking social support .78, avoidance .70).

The valid and reliable Multidimensional Scale of Perceived Social Support (MSPSS) was used to determine patients’ perceived social support. The 12 items were rated on a Likert scale from 1 (strongly disagree) to 7 (strongly agree). Total score was obtained by calculating the mean score for all items and ranged from 1 to 7. Higher scores indicate a higher level of perceived social support. In the current study, internal consistency was high (Cronbach alpha .84).

Quality of life (QoL) was assessed with the overall and general health domain of the abbreviated version of the World Health Organization Quality of Life assessment (WHOQOL-BREF) [23]. These two items were scored on a Likert scale from 1 (e.g., very unsatisfied) to 5 (e.g., very satisfied). Total scores ranged from 2 to 10, with higher scores indicating higher QoL. This instrument has good validity and reliability [24]. In the current study, internal consistency was high (Cronbach alpha .77).

### Statistical analysis

A step-3 latent profile analysis was performed in Latent Gold 5.1 [25] to examine the existence of a discrete number of different profiles based on a set of psychological characteristics (i.e., anxiety symptoms, depressive symptoms, avoidant coping, active coping, social support coping, and perceived social support) [25]. Several models were built by estimating models with an increasing number of profiles (i.e., 1-profile to 5-profiles model). Information criteria—the Bayesian information criterion (BIC), the AIC (Akaike Information Criterion), and the AIC3—were used to choose the most parsimonious and best fitting model [26]. The lower the BIC, AIC, and AIC3 values, the better a particular model is [25]. Additionally, content considerations and size of the subgroups that are reflected by the profiles were used to choose the best-fitting model [27].

To label the profiles, mean scores for all psychological characteristics within each profile were observed. For both anxiety and depressive symptoms, the cutoff ≥ 8 was maintained. For the coping styles and perceived social support, the median was used as the cutoff value. For all psychological characteristics, a score similar to or higher than the cutoff was interpreted as high, while scores below the cutoff were interpreted as low.

Next, it was evaluated whether there are significant differences between the identified psychological profiles regarding sociodemographic and medical characteristics, using multinomial logit models [25]. Two distinct models were tested: (1) sociodemographic model: age, sex (male/female), having a partner (yes/no), work status (employed/unemployed/retired), educational level (≤8 years/> 8 years) and (2) medical characteristics: smoking (currently or in the past/never), comorbidities (≥ 2 of the following: heart disease, stroke, high blood pressure, obstructive lung disease, diabetes, ulcer, kidney disease, liver disease, blood illness, thyroid disease, arthrosis, chronic back pain, rheumatism/< 2), small cell vs. non-small cell cancer, cancer stage (I, II, III, IV). Finally, the relationship between the identified profiles and QoL was evaluated in a third model. In all models, Wald statistics were used as a test of significance with a significance level of *p* < .05.

## Results

### Sample characteristics

Of the 130 patients who participated (mean age ± SD = 68.3 ± 8.6 years; 49% men), descriptive information on sociodemographic, medical, and psychological characteristics are presented in Table 1. Of the total sample, 113 (87%) were diagnosed with non-small cell lung cancer; 45% with stage I or II, 32% with stage III, and 23% with stage IV. The average HADS-Anxiety score was 5.4 ± 3.7; 34 (26%) patients scored above the clinical cutoff (≥ 8) and were classified as having significant anxiety symptoms. The average HADS-Depression scores were 5.0 ± 3.7; 34 (26%) patients scored above the clinical cutoff (≥ 8) and were classified as having significant depressive symptoms.

**Table 1:** Descriptive information on sociodemographic, medical, and psychological characteristics (*n* = 130)

Sociodemographic characteristics	% (*n*)
Age (years ± SD)	68.3 ± 8.6
Men	49 (64)
With partner^a^	75 (94)
Low education (≤ 8 years)^b^	21 (26)
Work status^a^	
Employed	12 (16)
Unemployed	37 (47)
Retired	51 (65)
Medical characteristics	*%* (*n*)
(History of) smoking	94 (122)
Comorbidities^1^	59 (77)
Non-small cell lung carcinoma	87 (113)
Cancer stage	
I-II	45 (58)
III	32 (42)
IV	23 (30)
Treatment^a^	
Surgery	45 (57)
Radiotherapy	41 (53)
Chemotherapy	55 (70)
Targeted therapy	6 (7)
Psychological characteristics	*Mean ± SD*
(History of) psychological treatment^a^ % (*n*)	20 (25)
Anxiety (HADS) (range 0–21)^a^ (cutoff ≥ 8)	5.4 **±** 3.7
Depression (HADS) (range 0–21)^b^ (cutoff ≥ 8)	5.0 ± 3.7
Coping (COPE)	
Active (range 8–32)^c^ (cutoff ≥ 24)	22.8 ± 5.7
Social support (range 6–24)^c^ (cutoff ≥ 14)	13.8 ± 3.7
Avoidant (range 6–24)^c^ (cutoff ≥ 11)	10.9 ± 3.4
Perceived social support (PSSS) (range1–7)^a^ (cutoff ≥ 6)	5.4 ± 1.2
Overall quality of life (WHOQOL-BREF) (range 2–10)^b^	7.0 ± 1.4

^a^1–4% missing values

^b^5–7% missing values

^c^8–11% missing values

^1^At least two of the following diseases: heart disease, stroke, high blood pressure, obstructive lung disease, diabetes, ulcer, kidney disease, liver disease, blood illness, thyroid disease, arthrosis, chronic back pain, rheumatism

*HADS*, Hospital Anxiety and Depression Scale; *COPE*, Coping Inventory for Stressful Situations; *PSSS*, Perceived Social Support Scale; *WHOQOL-BREF*, World Health Organization Quality of Life

### Four latent psychological profiles

The 4-profiles model was chosen; from Table 2, it can be observed that the BIC is lowest in the 3-profiles model, the AIC in the 5-profiles model, and the AIC3 in the 4-profiles model. Content of the profiles from the 3- to 5-profiles was compared. Also, the size of the subgroups that are reflected by the profiles was taken into account [27].

**Table 2:** Identification of the number of latent subgroups using regression models for psychological characteristics

Statistics					
Model	LL	Npar	BIC (LL)	AIC (LL)	AIC3 (LL)
1-profile	− 1886.15	12	3830.71	3796.30	3808.30
2-profiles	− 1810.04	25	3741.77	3670.08	3695.08
3-profiles	− 1777.84	38	3740.65*	3631.68	3669.68
4-profiles	*− 1749.81*	*51*	*3747.86*	*3601.62*	3652.62*
5-profiles	− 1732.67	64	3776.85	3593.33*	3657.33

The chosen model is presented in italics. Fit was evaluated evaluating the BIC, AIC, AIC3, and the size and content of the profiles as described in the Methods section. *lowest BIC, AIC, AIC3 value. *LL*, log likelihood; *Npar*, number of estimated parameters

**Fig. 1:**
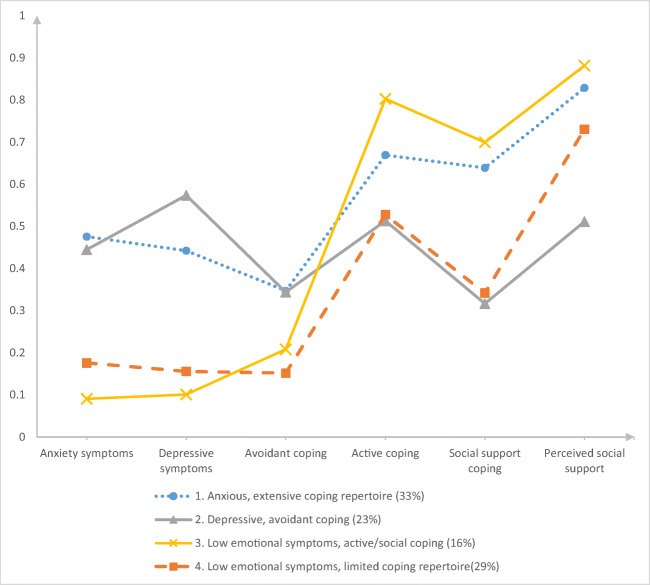
Latent psychological profiles of patients with lung cancer

To label the profiles, mean scores for all psychological characteristics (Table 3) within each profile were observed (Fig. 1).

- Profile 1: Anxious, extensive coping repertoire (33% of the total sample) was determined by high anxiety scores and low scores for depressive symptoms. Scores for all coping styles and perceived social support were high.
- Profile 2: Depressive, avoidant coping (23% of the total sample) was characterized by high scores for depressive symptoms and low scores for anxiety symptoms. While scores for avoidant coping were high, scores for active coping, social support coping, and perceived social support were low within this profile.
- Patients with profile 3: Low emotional symptoms, active/social coping (16% of the total sample) scored low on both anxiety and depressive symptoms. These patients scored low on avoidant coping, but high on the other coping styles and on perceived social support.
- Patients with profile 4: Low emotional symptoms, limited coping repertoire (29% of the total sample) reported low scores on all psychological characteristics.

**Table 3:** Mean scores for psychological characteristics within each profile

	1. Anxious, extensive coping repertoire	2. Depressive, avoidant coping	3. Low emotional symptoms, active/social coping	4. Low emotional symptoms, limited coping repertoire
	Mean			
Psychological characteristics (cutoff)				
Anxiety symptoms (≥ 8)	8*	7	2	3
Depressive symptoms (≥ 8)	7	9*	2	2
Avoidant coping (≥ 11)	12*	12*	10	9
Active coping (≥ 24)	24*	20	27*	21
Social support coping (≥ 14)	16*	11	17*	11
Perceived social support (≥ 6)	6*	4	6*	5

*≥ Cutoff

Cutoff for anxiety and depressive symptoms was ≥ 8, for the other characteristics the median was used, as described in the “Methods” section

### Sociodemographic and medical characteristics of the psychological profiles

Examining the set of sociodemographic characteristics, there were no significant differences between the psychological profiles regarding age (Wald = 2.85, *p* = .42), sex (Wald = .91, *p* = .82), work status (Wald = 4.67, *p* = .20), and educational level (Wald = 5.49, *p* = .14). Furthermore, no differences were found between the profiles in relation to medical characteristics, including a history of smoking (Wald = 4.12, *p* = .25), having comorbidities (Wald = .69, *p* = .87), small cell lung carcinoma (vs. non-small cell) (Wald = 1.25, *p* = .74), and cancer stage (Wald = 7.34, *p* = .29).

### QoL of the psychological profiles

**Fig. 2:**
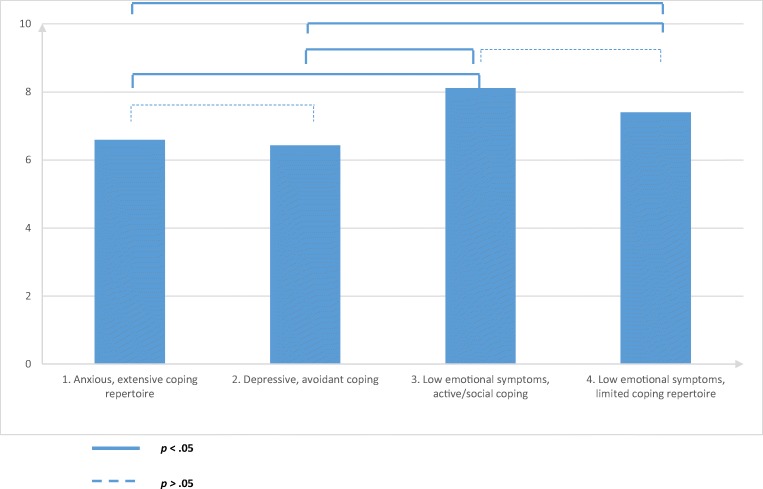
Psychological profiles and quality of life (QoL) for patients with lung cancer

An overall significant difference in QoL (Wald = 13.47, *p* = .003) was observed between the profiles (Fig. 2). An overview of the paired comparisons is presented in Table 4. QoL in profile 1: Anxious, extensive coping repertoire (QoL = 6.59) was significantly different from QoL in profile 3. Low emotional symptoms, active/social coping (QoL = 8.11) and profile 4. Low emotional symptoms, limited coping repertoire (QoL = 7.40). QoL in profile 2: Depressive, avoidant coping (QoL = 6.43) was also significantly different from QoL in profile 3. Low emotional symptoms, active/social coping (QoL = 8.11) and profile 4. Low emotional symptoms, limited coping repertoire (QoL = 7.40).

**Table 4:** Paired comparisons for quality of life (QoL)

Comparison			
First profile (QoL)	Second profile (QoL)	Wald	*p* value
1. Anxious, extensive coping repertoire (6.59)	2. Depressive, avoidant coping (6.43)	0.15	.70
1. Anxious, extensive coping repertoire (6.59)	3. Low emotional symptoms, active/social coping (8.12)	9.74	*.001*
1. Anxious, extensive coping repertoire (6.59)	4.Low emotional symptoms, limited coping repertoire (7.40)	6.40	*.01*
2. Depressive, avoidant coping (6.43)	3. Low emotional symptoms, active/social coping (8.12)	8.82	*.003*
2. Depressive, avoidant coping (6.43)	4. Low emotional symptoms, limited coping repertoire (7.40)	5.95	*.02*
3. Low emotional symptoms, active/social coping (8.12)	4. Low emotional symptoms, limited coping repertoire (7.40)	3.42	.07

*p* values < .05 are presented in italics

No significant difference was found between QoL in profile 1. Anxious, active extensive coping repertoire (QoL = 6.59) and profile 2. Depressive, avoidant coping (QoL = 6.43). Similar scores for QoL are also observed in profile 3. Low emotional symptoms, active/social coping and profile 4. Low emotional symptoms, limited coping repertoire (QoL = 8.11 and 7.40 resp.).

## Discussion

This study aimed to identify latent psychological profiles in patients with lung cancer, based on a broad set of psychological characteristics, including transient emotional symptoms (i.e., anxiety and depressive symptoms), relatively stable psychological factors (i.e., coping styles), and the extent of perceived social support. Additionally, it was examined how these profiles were associated with sociodemographic and medical characteristics and with QoL. Hypothesis 1 was confirmed; in patients with lung cancer, four psychological patient profiles were identified as follows: (1) Anxious, extensive coping repertoire; (2) Depressive, avoidant coping; (3) Low emotional symptoms, active/social coping; and (4) Low emotional symptoms, limited coping repertoire. There were no differences between the profiles regarding sociodemographic and medical characteristics, which rejected hypothesis 2. Hypothesis 3 was confirmed, as QoL was significantly different between the identified psychological profiles.

### Profile 1

Anxious, extensive coping repertoire reflected patients with anxiety symptoms in combination with having a broad repertoire of responses to deal with stressful situations, including active strategies (e.g., to make a plan), seeking social support (e.g., to ask someone for advice), and avoidance coping (e.g., to withdraw). Patients with this profile also perceived a high level of social support. Depressive symptoms were low in this profile but borderline; they almost succeeded the cutoff. A possible explanation may be that, in patients with this profile, anxiety symptoms function as a trigger, motivating to actively use various coping strategies to deal with the disease and its consequences [14]. This may be an attempt to increase the cancer-related self-efficacy or sense of control, which is not always helpful in this context, considering the fact that most patients with lung cancer are unable to “solve” their persisting disease by themselves (in terms of recovery or repair) [2, 28]. “Failing” at being able to gain control over the situation may therefore trigger avoidance as a second coping style (i.e., avoidant coping) and function as a sustaining factor with regard to the experienced anxiety and (below threshold) depressive symptoms.

### Profile 2

Depressive, avoidant coping was characterized by depressive symptoms in combination with an avoidant coping style. Anxiety symptoms were low in this profile, but scores almost succeeded the established cutoff. Patients in this profile had the tendency to withdraw (avoidant coping), rather than seeking support with family or friends (seeking social support coping) or trying to improve the situation (active coping). The level of perceived social support was low in this profile. Associations between avoidant coping and low levels of social support with depressive symptoms have been reported earlier [29, 30]. One of the mechanisms to explain this association may be that suffering from depressive symptoms may increase the tendency to withdraw or deny potential stressors. At the same time, avoidant coping strategies will not help to regulate negative emotions and may even increase the level of depressive symptoms. Thus, a vicious circle may occur: depressive symptoms (e.g., feeling sad) may lead to withdrawal from friends and family, which may increase the patients’ feeling of social isolation and lack of emotion regulation, reinforcing the depressive symptoms (in this case feeling sad) and perhaps even anxious symptoms.

### Patients with Profile 3

Low emotional symptoms, active/social coping reported low levels of emotional symptoms. These patients tended to use active coping strategies or seek social support rather than using avoidant coping strategies. Compared with the other profiles, patients with this profile also experienced the highest level of perceived social support. In earlier research, both active coping strategies and seeking social support have been associated with a lower risk of emotional symptoms, such as depression [29]. A possible explanation may be that active/social coping is more adaptive in patients with profile 3 than in profile 1*.* This may be explained by the fact that in this profile, it is not accompanied by high levels of avoidant coping. Overall, the effectiveness of a given coping style is context-dependent and subject to personality traits and circumstances [31]. In other words, the same coping style (in this case active/social coping) may be adaptive in one context (in this case profile 3) and maladaptive in another context (profile 1).

### Similar to patients with profile 3, patients with profile 4

Low emotional symptoms, limited coping repertoire reported low levels of emotional symptoms. However, their scores on all coping styles and on perceived social support were low, indicating limited reported responses to use in stressful circumstances. It may be that patients with this profile are able to accept their disease and the associated consequences [32]. However, another explanation for this profile may be that these patients maintain a more repressive coping style, which is defined as dismissing or ignoring strong emotions as self-protection and therefore did not report any emotional symptoms [33].

Compared with profile 3 and profile 4, QoL was lower in both profile 1 and profile 2, which fits previous findings showing strong relationships between anxiety, depressive symptoms, and a lower QoL [9, 13]. Interestingly, there were no significant differences between the profiles regarding sociodemographic and medical characteristics. This is in contrast with previous studies who did find positive correlations between age, sex, and/or cancer type with emotional symptoms [4, 9–11], but confirms the findings of Mitchell et al. (2011) and Hopwood et al. (2000) [6]. According to the current results, the combination of emotional symptoms, coping style, and the extent of perceived social support is stronger related to QoL than sociodemographic and medical characteristics. This is in line with the studies who found that specific psychological factors were stronger related to depressive symptoms and QoL as compared with sociodemographic and medical characteristics [12, 13].

### Scientific implications

The current results are useful for further scientific research examining the association between psychological profiles and QoL. Considering patient’s psychological profiles, covering information about emotional symptoms, coping style and the extent of perceived social support, instead of just the presence/absence of anxiety or depressive symptoms, might add important explanatory and predictive power.

### Clinical implications

The current results may be useful in clinical practice. For example, during the process of shared decision-making (SDM) [34]. SDM is defined as the process in which patients’ values and personal preferences are routinely integrated in clinical care, by means of patient-centered conversations [35]. Being aware of patients’ psychological profiles may optimize the quality of these conversations, as it may broaden the scope of the conversation more explicitly. Early identification of patients’ psychological profiles may also help in more adequately selecting the patients who are in need of psychological treatment, as profiles provide unique information that is not well covered by the use of scores on single psychological factors [36]. Furthermore, these profiles may be useful in aligning care with patients’ unique preferences and needs as this unique information may help in choosing the target of psychological intervention. While the presence of emotional symptoms and the coping strategies used is often involuntary, application of ways of coping can be trained, e.g., by cognitive behavioral therapy given by a specialized professional, such as a medical psychologist [37]. Strengthening patients’ resources may improve outcomes as QoL [2] and more adequate decision-making.

Furthermore, it may be hypothesized that these profiles may explain certain patient behaviors. For example, non-compliance with medication or other medical advises may result from a combination of depressive symptoms and a passive coping style, as in patients with profile 2. These patients may need more encouragement during their treatment, as compared with patients with profile 1 (Anxious, extensive coping repertoire). Additional research in this regard is however needed.

### Study strengths and limitations

The current results need to be interpreted in light of the study’s strengths and limitations. To our knowledge, this is the first study from a more person-centered approach, examining combinations of multiple psychological, sociodemographic, and medical variables, including transient psychological symptoms (e.g., depressive symptoms) and more stable traits (e.g., coping style) in this population. However, all psychological variables were assessed with self-report instruments, which may not completely reflect actual psychological functioning and behaviors. Another shortcoming of this study was the under-representation of patients with stage IV and small cell lung cancer. An explanation for this fact is the limited life expectancy and rapid deterioration of mental and bodily functioning in patients with stage IV and small cell lung cancer, which understandably interferes with study compliance. Moreover, the current study had a cross-sectional design, which makes it impossible to draw conclusions about causal relationships. For future research, it may be considered relevant to include a clinical evaluation on psychological functioning, e.g., in the form of a structured clinical interview, to evaluate if they confirm the results found with self-report questionnaires. Furthermore, it may be interesting to prospectively study the association of psychological profiles with other (long-term) outcomes (e.g., treatment adherence and survival) and cancer-induced/related toxicity.

## Conclusion

In conclusion, the current results show the importance of determining the psychological profiles of patients with lung cancer in an early stage. Insight in psychological profiles will help in selecting those patients who are in need of psychological screening and/or psychological treatment. This information is useful for the process of SDM, which aims to individualize the treatment process to patients’ unique characteristics and preferences. Furthermore, the current findings highlight the potential role for specialized caregivers, such as a medical psychologist, in the treatment process of patients with lung cancer.
